# Supplementary material for: Amikacin Coated 3D-Printed Metal Devices for Prevention of Postsurgical Infections (PSIs)
Source: Pharmaceutics. 2025 Jul 14;17(7):911. doi: 10.3390/pharmaceutics17070911 (PMC12298719; doi:10.3390/pharmaceutics17070911)
Supplement: Supplementary file 1 [file pharmaceutics-17-00911-s001.zip › pharmaceutics-3741842-supplementary.pdf]

pharmaceutics

Supplementary Materials: Amikacin Coated 3D-Printed Metal Devices for Prevention of Postsurgical Infections (PSIs)

Chu Zhang [1], Ishwor Poudel, Nur Mita, Xuejia Kang, Manjusha Annaji, Seungjong Lee, Peter Panizzi, Nima Shamsaei, Oladiran Fasina, R. Jayachandra Babu and Robert D. Arnold

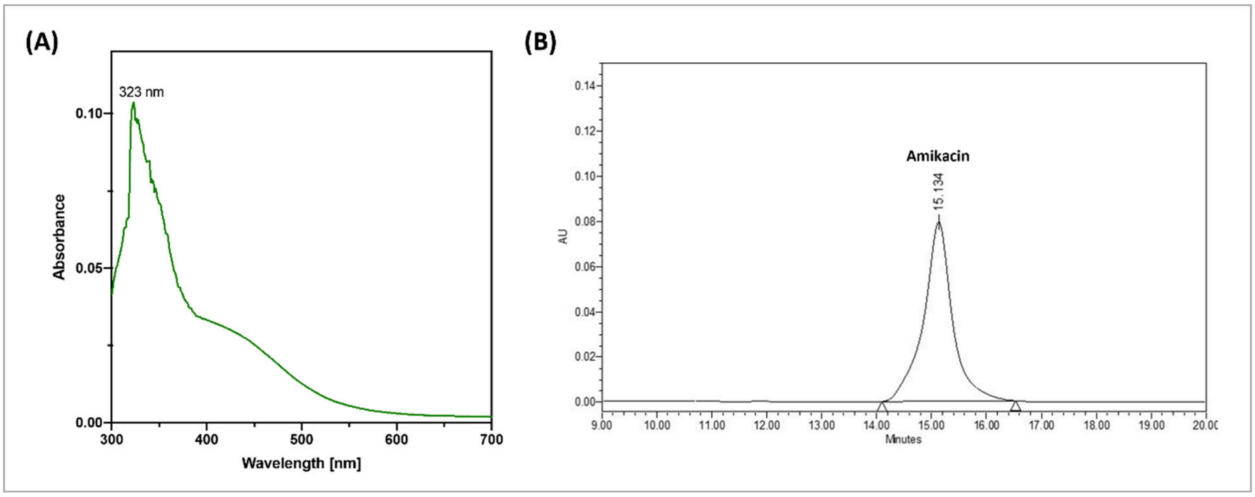

Figure S1. (**A**) UV-Visible spectrum and (**B**) Chromatogram of an amikacin standard solution (80 μg/mL) after derivatization with FDNB reagent.

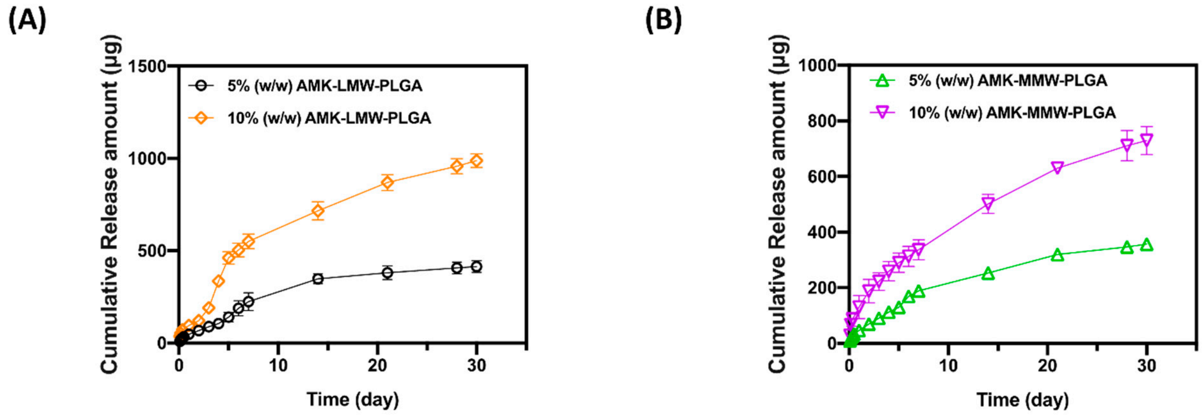

Figure S2. Cumulative release amount of amikacin from LMW chitosan (**A**) and MMW chitosan (**B**) with PLGA layer coatings. (n = 6).

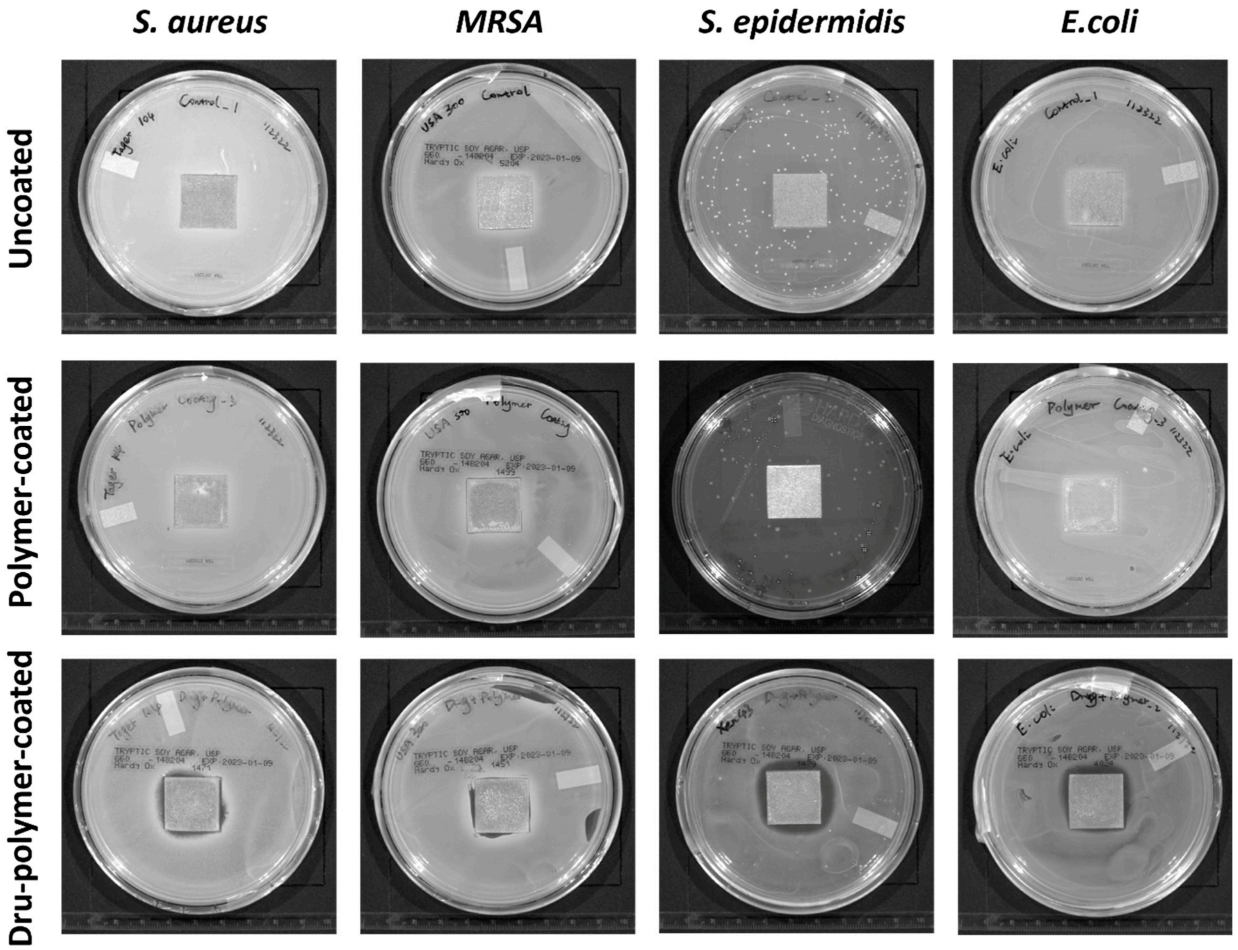

Figure S3. Zone of Inhibition was observed for uncoated, polymer-coated, and drug-polymer-coated implants placed in direct contact with a freshly streaked agar plate.

Table S1. Development of HPLC methods used for amikacin analysis.

HPLC conditions	Details
Mobile phase	Solvent A: 0.1% acetic acid in water<br>Solvent B: Acetonitrile
Column	Phenomenex Luna 5µ C_{18} column<br>(250 × 4.60 mm, i.d. 5µm)
Running program	55% solvent A, 45% solvent B
Total Running Time	20 minutes
Retention Time	Amikacin: 14.8-15.4 min
Flow rate	1 mL/min
Autosampler temperature	25°C
Injection Volume	10 µL
Column Temperature	45°C
Detector wavelength	323 nm

Table S2. Preparation of an amikacin calibration curve.

Final analyte Concentration (µg/mL)	200 µg/mL of amikacin stock (µL)	1 mg/mL of amikacin stock (µL)	PBS (µL)	Methanol (µL)	5 mg/mL of NaOH (µL)	180 mg/mL of FDNB (µL)
0.5	2.5	-	97.5	700	100	100
1	5	-	95	700	100	100

5	25	-	75	700	100	100
10	50	-	50	700	100	100
20	100	-	-	700	100	100
40	-	40	60	700	100	100
80	-	80	20	700	100	100
100	-	100	-	700	100	100

Table S3. Linearity and sensitivity of the proposed HPLC method.

Parameters	Values
Calibration range	0.5-100 μg/mL
Linear regression equation (*Y*)	48415*X*–3725.9
Regression coefficient (R^2)	0.999
p-value	< 0.0001
Limit of detection (LOD)[a]	0.025 μg/mL
Limit of quantification (LLOQ)[b]	0.5 μg/mL

X-concentration of amikacin (μg/mL); *Y*-peak area of amikacin derivative, [a] $S/N = 3.3$, $n = 6$, [b] $S/N = 10$, $n = 6$

Table S4. Evaluation of the accuracy of the proposed HPLC method.

Concentration (μg/mL)		Recovery (%) [a]	R.S.D. (%) (*n*=3)
**Added**	**Recovered**		
5.00	4.99	99.9	
5.00	5.01	100.1	0.27
5.00	5.02	100.4	
40.00	39.97	99.9	
40.00	40.23	100.6	0.89
40.00	40.68	101.7	
80.00	82.12	102.6	
80.00	82.78	103.5	1.01
80.00	81.14	101.4	

[a] (Found concentration/added concentration) × 100

Table S5. Evaluation of the intra- and inter-day precision of the proposed HPLC method.

Concentration (μg/mL)	R.S.D. (%) (*n* = 6)	
	**Intra-day**	**Inter-day**
5.00	5.29	11.01
40.00	7.24	11.26
80.00	3.98	11.79

Table S6. Evaluation of the robustness of the proposed HPLC method.

Parameters	Values	Recovery (%)	R.S.D. (%)
	0.9	106.7	0.62
Flow rate (mL/min)	1.0	100.0	6.73
	1.1	107.4	1.50
	43	97.1	7.51
Mobile phase (% acetonitrile)	45	100.0	6.73
	47	103.6	5.73
	43	103.0	8.09
Column temperature (± 0.5°C)	45	100.0	6.73
	47	103.5	6.87
